# Chronic Unilateral Idiopathic Trochleitis Misdiagnosed With Migraine in an 18-Year-Old Male: A Case Report

**DOI:** 10.7759/cureus.45010

**Published:** 2023-09-11

**Authors:** Walid M Abdalla, Rawan Alhalabi

**Affiliations:** 1 Ophthalmology, Orbit Eye Center, Dubai, ARE; 2 Pediatrics, American Hospital Dubai, Dubai, ARE

**Keywords:** blurred vision, diplopia, migraine, eye pain, trochleitis

## Abstract

Trochleitis is a very rare form of inflammation that occurs in the trochlear region. It is characterized by periorbital pain, diplopia, blurred vision, frontal headache, trochlear tenderness, and radiologic signs of inflammation. We report a case of an 18-year-old man who experienced unilateral eye pain, double vision, and tenderness when looking upward. Initially, his pain was misdiagnosed as migraine for more than two months. The patient did not show improvement with NSAID treatment, leading to successful treatment with a steroidal injection. Although rare, healthcare providers should maintain a high index of suspicion to avoid misdiagnosis of this type of eye pain.

## Introduction

Idiopathic trochleitis is a highly uncommon disease that mostly involves pain in the inner angle of the orbit, accompanied by signs of inflammation in the superior oblique tendon, and trochlear apparatus [1]. Typically, it presents with severe intermittent orbital pain triggered by vertical eye movements or reading [2,3]. Other manifestations may include trochlear tenderness, diplopia, blurred vision, ipsilateral frontal headache [2-4], and, on rare occasions, restricted eye movement. Diagnosis generally relies on the clinical picture and radiologic evidence of inflammation [1,2]. Herein, we present an exceedingly rare case of an 18-year-old male who experienced persistent left eye pain, initially misdiagnosed as a migraine for over two months.

## Case presentation

An 18-year-old man presented to the clinic due to recurrent left orbital pain and ipsilateral forehead headache over two months. He had been in good health until two months prior when he experienced his first severe pain episode. At that point, he was diagnosed with migraine and was prescribed ibuprofen and diclofenac elsewhere. While the pain improved to some extent, it did not completely subside. The pain was exacerbated when reading or looking upward, causing his vision to become blurry, with the appearance of two overlapping lines. There was no reported history of trauma or upper respiratory tract infection, and his family history was non-contributory.

Upon examination, his visual acuity was 20/20 in both eyes, and there was significant localized tenderness over the trochlear region. Full extraocular motility was observed. During the cover-uncover test, the patient exhibited orthophoric alignment in all primary positions except for downward gaze, where he displayed a left hyperopia of 4 degrees.

**Figure 1:**
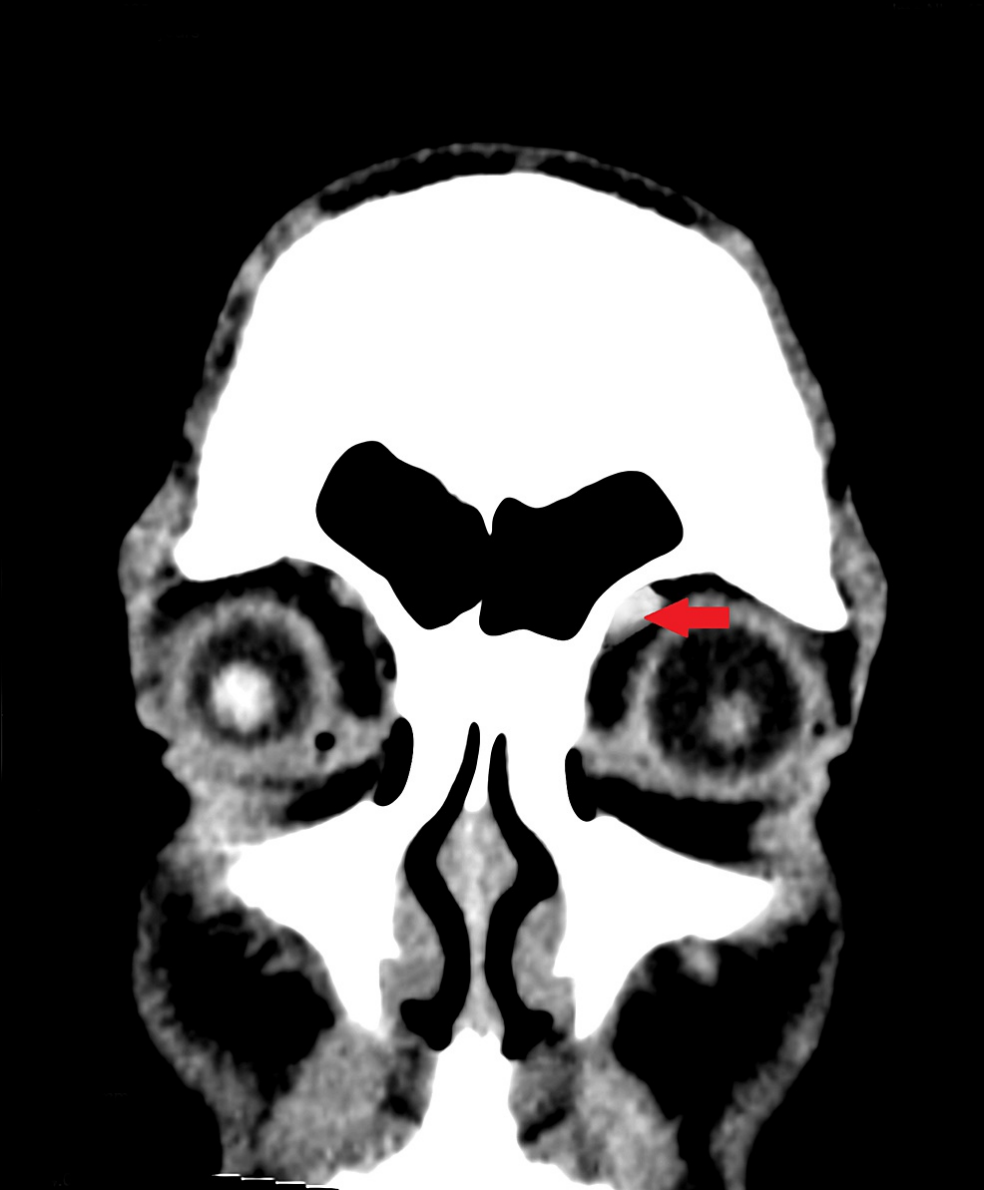
CT scan showing enlargement of the left superior oblique pulley and tendon (red arrow) CT, computerized topography

Lab tests for rheumatoid arthritis (RA) and systemic lupus erythematosus (SLE) were negative. Figure 1 depicts a computerized topography (CT) scan that revealed slight swelling on the left trochlear lesion along with mild enlargement of the superior oblique pulley and tendon, indicating trochleitis. Otherwise, no abnormalities were detected.

The treatment involved a single shot of combined 0.5 cc of lidocaine 2%, 0.25 cc of short-acting steroid (dexamethasone), and 0.5 cc of long-acting steroid (methylprednisolone), using a 27-gauge needle in the trochlear area, without penetrating any blood vessel. He reported mild pain relief and no double vision within three to four days of the injection.

At a three-month follow-up visit, he stated complete pain relief with no blurry vision or vertical diplopia. During the cover-uncover test, he exhibited orthophoria in all gaze positions with a left hypertrophic flick in a downward gaze.

## Discussion

Reported cases regarding the clinical characteristics and management of trochleitis are limited. It was first identified in 1984 by Tychsen et al., who studied 13 patients diagnosed with trochleitis [4]. Subsequently, a few papers reported on an additional 118 patients in both adult and pediatric age groups [1-3,5-7].

All the authors emphasized that trochleitis demonstrates a constellation of features: orbital pain aggravated by vertical eye movement, especially adduction, tenderness in the central area of the trochlea, and signs of inflammation, including swelling of the superior oblique muscle [1-8]. Our patient exhibited all of these features. Nonetheless, he did not have any previous history of trauma, upper respiratory tract infections, or migraine that provoked his trochleitis. 

Moreover, diplopia, gaze restriction, and ptosis were rarely encountered in the reported cases [2,3]. We hypothesize that our patient experienced vertical diplopia in the downward gaze, possibly due to inflammation of the superior oblique muscle.

Brown syndrome is a rare disease identified as trochleitis provoked by systemic inflammatory illnesses or trauma [3]. Systemic trochleitis is invariably associated with diplopia and restricted eye movement [3,5]. Although our patient’s medical history and blood tests excluded any systemic inflammatory process, he might develop symptoms later on.

Currently, there are no established guidelines for the treatment of trochlear pain. Generally, NSAIDs are not effective. A significant number of reported cases required systemic or local steroids [2,3,5]. A retrospective study conducted by Jarrín et al. suggested that NSAIDs are effective when pain is the sole symptom [3]. The most effective treatment primarily involved injectable short-acting or long-acting corticosteroids [2,3,5,6]. Similarly, in our case, NSAIDs did not alleviate the pain. Giannaccare et al. recommended serial steroidal injections as a second-line management option after NSAIDs. Surgical intervention is indicated if medical treatment proves ineffective [5].

## Conclusions

While rare, trochleitis should be considered when assessing periorbital pain in both adults and pediatrics. Healthcare providers should maintain a high index of suspicion to avoid misdiagnosis. We recommend evaluating for trochleitis in every patient experiencing prolonged, severe unilateral eye pain, fuzzy vision, or vertical diplopia.
